# Genome Sequence of the Native Apiculate Wine Yeast *Hanseniaspora vineae* T02/19AF

**DOI:** 10.1128/genomeA.00530-14

**Published:** 2014-05-29

**Authors:** Facundo M. Giorello, Luisa Berná, Gonzalo Greif, Laura Camesasca, Valentina Salzman, Karina Medina, Carlos Robello, Carina Gaggero, Pablo S. Aguilar, Francisco Carrau

**Affiliations:** a Departamento de Ecología y Evolución, Facultad de Ciencias, Universidad de la República, Montevideo, Uruguay; b Sección Enología, Facultad de Química, Universidad de la República, Montevideo, Uruguay; c Unidad de Biología Molecular, Institut Pasteur de Montevideo, Montevideo, Uruguay; d Departamento de Biología Molecular, Instituto de Investigaciones Biológicas Clemente Estable, Montevideo, Uruguay; e Laboratorio de Biología Celular de Membranas, Institut Pasteur de Montevideo, Montevideo, Uruguay; f Departamento de Bioquímica, Facultad de Medicina, Montevideo, Uruguay

## Abstract

The use of novel yeast strains for winemaking improves quality and provides variety including subtle characteristic differences in fine wines. Here we report the first genome of a yeast strain native to Uruguay, *Hanseniaspora vineae* T02/19AF, which has been shown to positively contribute to aroma and wine quality.

## GENOME ANNOUNCEMENT

Even though *Saccharomyces cerevisiae* produces most of the ethanol in wine, apiculate yeasts of the genus *Hanseniaspora* are the main species present on grapes and they play a significant role at the beginning of fermentation (1–3). The genus *Hanseniaspora* includes at least six species associated in two groups (4): *valbyensis, guilliermondii, uvarum* and *vineae, osmophila, occidentalis*. We have shown (5) that wines produced by co-fermentation of *H. vineae* and *S. cerevisiae* consistently exhibit more intense flavors and complexity and are significantly more full-bodied than wines produced by *S. cerevisiae*. Indeed, the co-fermentation strategy with *Hanseniaspora* species provided significant increases in glycerol and acetate ester flavor compounds and relative decreases in higher alcohols and fatty acids which correlates with the wine differences found between these alternative fermentation procedures (5). Thus, it will be of particular interest to characterize the genes differentially associated with these processes. We present the genome of *H. vineae* T02/19AF in order to contribute to a better understanding of its “flavor phenotype.”

*H. vineae* T02/19AF was isolated from Tannat wine fermentation, the typical red grape of Uruguay (6). Sequencing was performed on an Illumina Genome Analyzer IIx platform and generated 13,302,566 paired-end reads (2×100 cycles) representing an average coverage of 212-fold. Reads were filtered and trimmed with QC Toolkit (7), and redundancies were removed using Trinity *in silico* normalization (8). The processed reads were then assembled using MaSuRCA (9) (cgwErrorRate=0.15, insert size=900). Based on reciprocal BLASTn (10), redundant contigs (those included in a bigger read) were removed. A final assembly of 277 contigs (>500 pb) was obtained, which formed 124 scaffolds with a total length of 11,401,444 bp. The genome has an *N*_50_ of ~261 kb with an average G+C content of 37%, very similar to *S. cerevisiae* (11, 12).

A total of 4,733 putative open reading frames (ORFs) >100 nucleotides were predicted using Augustus (13) trained with *S. cerevisiae*. Automatic gene annotation using BLASTp (10) revealed that 4,206 ORFs (89%) are homologous to sequences of the NCBI’s non-redundant protein database from which 3,879 had at least one Pfam domain, indicating the high reliability of the predictions. Moreover, 4,061 predictions presented homology with 3,849 *S. cerevisiae* S288C strain distinct genes.

One hundred twenty eight genes associated with fermentation, such as those participating in glycolysis/gluconeogenesis, citrate cycle, pentose pathway, steroid biosynthesis, fatty acid degradation, and fatty acid biosynthesis pathways (14), from KEGG (15) were analyzed. Despite the great sequence divergence observed between *H. vineae* and *S. cerevisiae*, 87 of those genes (68%) were found through BLASTp in the genome of *H. vineae* T02/19AF. The accurate analysis of these genes will help further the understanding of the “flavor phenotype” of this and other yeast species. To the best of our knowledge, this is the first report of a *Saccharomycodaceae* yeast family genome.

### Nucleotide sequence accession numbers.

This whole-genome shotgun project has been deposited at DDBJ/EMBL/GenBank under the accession no. JFAV00000000. The version described in this paper is version JFAV02000000.
